# Nudging Toward Sustainable and Healthy Diets: A Randomized Trial of Young Adults

**DOI:** 10.1016/j.cdnut.2026.109384

**Published:** 2026-06-01

**Authors:** Carmen E Prestemon, Anna H Grummon, Sarah M Frank, Checkna Diawara, Lindsey Smith Taillie

**Affiliations:** 1 Department of Nutrition, Gillings School of Global Public Health, University of North Carolina at Chapel Hill, Chapel Hill, NC, United States; 2 Carolina Population Center, University of North Carolina at Chapel Hill, Chapel Hill, NC, United States; 3 Department of Pediatrics, Stanford University School of Medicine, Palo Alto, CA, United States; 4 Department of Health Policy, Stanford University School of Medicine, Stanford, CA, United States

**Keywords:** choice architecture, climate change, front-of-package label, food policy, dietary interventions

## Abstract

**Background:**

Young adulthood is an optimal time for intervening in dietary behaviors. Young adults in the United States strongly value environmental sustainability, so appealing to these values could be a promising strategy to improve dietary quality.

**Objectives:**

We examined whether a “stealth” intervention that explicitly appealed to sustainability (climate-impact labels, climate-focused social comparison messages) while also promoting healthier options (through healthy and sustainable swap recommendations) improved the healthfulness of food selections.

**Methods:**

Participants (United States young adults aged 18–25 y) were randomized in Qualtrics to the experimental (*n* = 1074) or control (*n* = 1075) arm and selected 1 protein, frozen meal, and snack they would most like to purchase. All foods varied by healthfulness (Nutri-Score algorithm, range A–E). Participants in the experimental arm received nudges appealing to sustainability, including viewing front-of-package labels showing whether the food had low, medium, or high climate-impact and, if they selected a high climate-impact food, viewing a climate-focused social comparison message and swap recommendation to low or medium climate-impact foods with a healthy Nutri-Score (A or B). Control arm participants did not receive any nudges.

**Results:**

Experimental arm participants selected foods that were 3.9 points healthier than participants in the control arm (*P* < 0.001, *d* = 0.67). Effects of the nudges on healthfulness did not differ by gender, education, financial situation, red meat consumption, political beliefs, or interest in sustainability (all *P*-interaction ≥0.13). Experimental arm participants selected foods with a carbon footprint 0.76 kg CO_2_-eq/100 g lower than control arm participants (*P* < 0.001, *d* = 0.93). Compared with the control, experimental arm participants thought more about sustainability (*P* < 0.001), less about taste (*P* < 0.001) and cost (*P* = 0.02), and similar amounts about healthfulness (*P* = 0.32) when selecting foods.

**Conclusions:**

“Stealth” nudges appealing to sustainability while also incorporating health considerations could encourage a wide range of young adults to select more healthful and sustainable foods.

This trial was registered at clinicaltrials.gov as NCT06119165 and at aspredicted.org as 147,997.

## Introduction

Poor dietary quality is a leading cause of noncommunicable chronic disease, including cardiovascular disease, type 2 diabetes, and some cancers [1]. Diet quality is especially poor among young adults in the United States, who have lower quality diets than middle-aged and older adults [[2], [3], [4]]. This is concerning because dietary behaviors in young adulthood are predictive of health risks at older ages, including metabolic syndrome, insulin resistance, and cardiovascular disease [5,6]. Young adulthood is also an optimal time for interventions to improve diet quality because it is a period of life associated with major changes in dietary behaviors, including reduced intake of fruits and vegetables and increased intake of sugary drinks [[7], [8], [9]]. Improving diet quality among young adults is therefore a promising strategy for reducing the burden of chronic disease.

Evidence suggests that health-focused interventions have the potential to improve dietary quality [10,11]. However, health-focused interventions miss an opportunity to appeal to other motivating values, such as environmental sustainability. Young adults in the United States are supportive of environmental sustainability [12,13], though they may lack knowledge on sustainable behaviors [14]. Appealing to their interest in sustainability could therefore be a promising strategy for encouraging young adults to change their diet [15]. There is considerable overlap between environmentally sustainable dietary patterns and healthful dietary patterns—for example, both encourage consumption of plant-based foods like fruits, vegetables, nuts, and legumes and discourage consumption of animal-based foods like red and processed meat [16,17]. Thus, encouraging sustainable eating is likely to have the added benefit of also improving diet quality.

One promising avenue for appealing to young adults’ interest in sustainability is through nudges—a form of choice architecture that primes consumers to make small changes without punishment or reward [18,19]. In the context of food selection, the shopping environment may be designed in a manner that makes an item more likely to be selected [20]. For example, nudges to encourage sustainable food choices could include front-of-package labels that communicate a food’s climate impact, social comparison messages that compare one’s climate-related behaviors to peers, and swap recommendations that offer consumers a recommendation to switch from a high climate-impact to a low or medium climate-impact food. Sustainability-focused nudges have been shown to increase pro-environmental behaviors [[21], [22], [23], [24]] and health-focused nudges have been found to lead consumers to buy healthier foods [[25], [26], [27], [28], [29]]. Given young adults’ interest in sustainability, it is possible that implementing sustainability-focused nudges could encourage them to buy more sustainable foods. If those sustainability nudges were designed to also promote healthy foods (e.g., by only offering swaps to foods that are *both* low or medium climate-impact and healthier), interventionists might be able to “stealthily” improve young adults’ diet quality despite only explicitly appealing to sustainability [15]. This possibility, however, has not been evaluated.

To address these gaps, this study aimed to examine the impact of a stealth intervention that explicitly appealed to sustainability while also incorporating health considerations (in the form of 3 nudges: climate-impact labels, climate-focused social comparison messages, and healthy and sustainable swap recommendations) on the healthfulness of food selections. We hypothesized that exposure to the suite of nudges would improve the healthfulness of young adults’ food selections.

## Methods

### Participants

From 28 November, 2023 to 4 December, 2023, we recruited a convenience sample of 2575 young adult participants using CloudResearch Prime Panels. CloudResearch Prime Panels is a market research panel frequently used for online research [30]. Participants were eligible if they were between the ages of 18 and 25 and currently residing in the United States. CloudResearch targeted recruitment to match the Census distribution of race/ethnicity and gender for this age group. To account for eligibility requirements for an auxiliary study, we used quotas to ensure that ≥25% of the study sample was enrolled full-time in college [31].

### Procedures

Participants completed an online survey programmed in Qualtrics. After responding to eligibility questions and completing the electronic informed consent, participants were randomized in a 1:1 simple allocation ratio in Qualtrics to the experimental arm (*n* = 1074) or the control arm (*n* = 1075). All participants then completed a food selection task, where they were instructed to select one food that they would most like to purchase within each of 3 different food categories: proteins, frozen meals, and snacks. We focused on these categories because they are top contributors to dietary greenhouse gas emissions (GHGE) in the United States [32]. After selecting their preferred foods in each category, participants responded to questions about the foods and labels, as well as standard sociodemographic questions.

#### Products shown in the food selection task

We selected 17 proteins, 17 frozen meals, and 17 snacks for the selection task. We selected products to ensure that we represented a range of types of foods in each category (e.g., the protein category included chicken, beef, pork, seafood, and vegetarian options) as well as a range of options in terms of healthfulness and sustainability. Specifically, we aimed to include products according to the main subcategories listed by a major United States online grocery store. For example, we aimed to include the following products in the snack category: chips, pretzels, and popcorn; cookies; nuts and dried fruit; and snack meats and jerky, as these were the main subcategories in the “Snack” department of the major United States online grocery store. Within subcategories, we sorted products by popularity in the online grocery store and included the most popular products while ensuring a variety of brand representation. After the development of an initial product list, we further incorporated vegetarian options (if they were not already present). Finally, we ensured that each category had ≥3 healthy and sustainable products (defined as a Nutri-Score of A or B and in the lowest tertile of carbon pollution; see *Intervention* and *Primary outcomes* sections for more details on product scoring).

#### Intervention

Participants in the experimental arm were exposed to a suite of sustainability-focused nudges during the selection task, including climate-impact labels, social comparison messages that compared a participant’s selection to that of “sustainable shoppers” (“climate-focused social comparison messages”), and healthy and sustainable swap recommendations. We included climate-impact labels in the suite of nudges because research indicates that front-of-package labels improve the healthfulness of food selections [33]. We included climate-focused social comparison messages because studies have found that consumers are motivated to align with social norms when making purchasing decisions [34]. Finally, we included swap recommendations because randomized trials indicate that they encourage healthier food purchases [28,29,35]. All participants in the experimental arm saw the climate-impact labels. Participants in the experimental arm who selected a high climate-impact food were then shown climate-focused social comparison messages and healthy and sustainable swap recommendations as described below. Participants in the experimental arm who selected a low or medium climate-impact food were not shown additional interventions.

**FIGURE 1:**
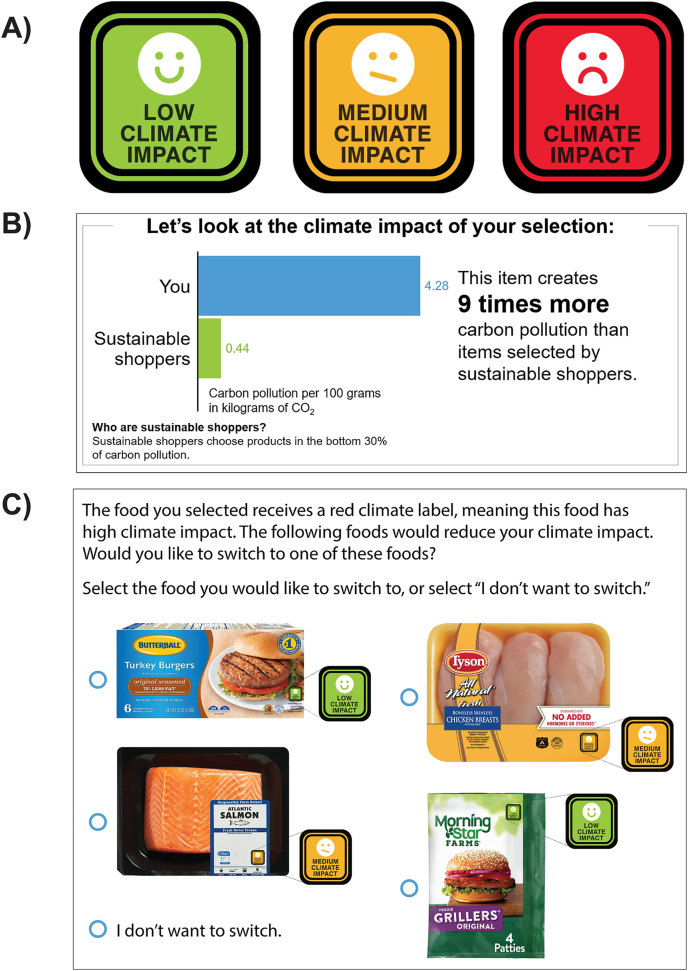
Interventions. (A) Climate-impact labels. (B) Sample climate-focused social comparison message. (C) Sample healthy and sustainable swap recommendations.

The climate-impact labels are shown in Figure 1A. The climate-impact labels were color-coded and had text and emoticons to communicate the climate impact of each product, similar to the traffic light labeling system [33] and labels from a previous nutrition labeling study [36]. Specifically, high climate-impact labels were red with a frowning face emoticon; medium climate-impact labels were yellow with a neutral face; and low climate-impact labels were green with a smiley face. The labels used color and emoticons because research finds that these design elements may enhance the effectiveness of labels [37,38]. We assigned labels to foods based on the carbon emissions associated with producing that food [in terms of kilograms of carbon equivalents (kg CO_2_-eq) per 100 g of product]. We estimated each food’s carbon footprint using data from the database of Food Recall Impacts on the Environment for Nutrition and Dietary Studies (dataFRIENDS) [39,40]. The dataFRIENDS methodology has been described in detail by Heller, Rose, and colleagues [41,42].

Briefly, researchers first developed the Database of Food Impacts on the Environment for Linking to Diets (dataFIELD) by calculating the GHGE associated with each of the 332 commodities in the Food Commodity Intake Database (FCID) from seed to farm gate [41,42]. GHGE data are reported in kg CO_2_-eq per edible 100 g of food. Researchers then developed the dataFRIENDS database by linking the FCID with NHANES foods [39,40]. In the present study, 2 trained research assistants matched each food to its closest NHANES food commodity and corresponding GHGE in the dataFRIENDS database (see Supplemental Methods for more details). Foods offered in the selection task had median GHGE of 0.37 kg CO_2_-eq/100 g (IQR: 0.16–0.79 kg CO_2_-eq/100 g) (Supplemental Table 1). Foods in each category were divided into tertiles of high, medium, or low carbon pollution, and the corresponding climate-impact labels were applied to the package.

If a participant selected a low or medium climate-impact product, they were not exposed to any additional interventions. If the participant selected a product with a high climate-impact label, they were then shown a climate-focused social comparison message and asked if they would like to swap their selection. The climate-focused social comparison messages compared the participant’s selection to that of “sustainable shoppers,” similar to messaging used by electricity companies to communicate how a household’s energy usage compares with their energy-efficient neighbors [43] (see Figure 1B for a sample message). Evidence suggests that home energy reports are an effective method to reduce household energy consumption [44,45]. We adapted the message text and visual design from a sample Opower home energy report [46,47]. A graphic designer created the climate-focused social comparison messages, and all members of the research team approved the final product. We determined values for the “sustainable shoppers” by averaging the carbon emissions of the low and medium climate-impact products within each category. After viewing the climate-focused social comparison message, the participant was asked if they would like to swap their high climate-impact selection to 1 of 3 to 4 low or medium climate-impact foods, similar to a previous experiment [29] (Figure 1C). The low or medium climate-impact foods offered to participants were from the pre-existing choice set. Although the messages accompanying the swaps appealed only to sustainability, we set a rule that all potential swaps had to be healthy, defined as having a Nutri-Score of A or B (see below for details on scoring). This approach mirrors previous studies [[27], [28], [29]] and offers a way to “stealthily” nudge healthier selections without explicitly invoking health considerations [15]. Participants were also given the opportunity to keep their original high climate-impact selection.

Participants in the control arm selected foods without viewing climate-impact labels, climate-focused social comparison messages, or healthy and sustainable swap recommendations.

### Ethics statement

Study procedures were reviewed and determined exempt from further review by the University of North Carolina at Chapel Hill Institutional Review Board (#23-1785). All study participants provided electronic informed consent. Participants were compensated for their participation with their preferred form (gift cards, rewards points, or money) by CloudResearch. CloudResearch’s Prime Panels aggregates multiple market research panels, so the exact incentive amount cannot be quantified [48].

### Measures

#### Primary outcome

The primary outcome was the healthfulness of product selections, operationalized using Nutri-Score scores. We calculated scores using the Nutri-Score algorithm updated in 2022 [49]. Briefly, the algorithm assigns summary scores to foods based on nutrients and ingredients to consume compared with those to limit according to health recommendations. Numerical scores ranging from −15 to 40 are assigned to products, with a *lower* score indicating a more healthful product [49]. For this study, to improve interpretability, we inverted the Nutri-Score, with a *higher* Nutri-Score indicating a more healthful food. The Nutri-Score algorithm is described in detail elsewhere [49]; additional methodological details are also provided in the Supplemental Methods. Nutri-Scores of foods offered in the food selection task had a median score of 21 (IQR: 12–25).

#### Secondary outcomes

The secondary outcome from the food selection task was the carbon footprint of product selections, operationalized at the product’s GHGE expressed as carbon dioxide equivalents (i.e., kg CO_2_-eq/100 g) (see above and Supplemental Methods for more details). The survey measured additional secondary outcomes (Supplemental Table 2). First, we measured cognitive elaboration, or how much the participants thought about the environmental sustainability, health, taste, and cost of the products while completing the food selection task. This was assessed with the question, “We are interested in how you selected foods. When you selected foods, how much did you think about each of the following characteristics? Environmental sustainability, health, taste, and cost.” Response options ranged from 1 (Not at all) to 5 (A great deal).

Next, we assessed perceived sustainability, perceived healthfulness, and purchase intentions of sustainable and unsustainable products. Participants viewed and rated 1 sustainable (i.e., low climate-impact) and 1 unsustainable (i.e., high climate-impact) product (selected at random from among those shown in the food selection task) from 1 of the 3 food categories (i.e., proteins, frozen meals, or snacks; selected at random). Perceived sustainability was assessed with the question, “How environmentally sustainable do you think this food is?” Perceived healthfulness was assessed with the question “How healthy do you think this food is?” Both perception questions used response options ranging from 1 (Not at all) to 5 (Extremely). Purchase intentions were assessed with the question, “How likely would you be to buy this food in the next month, if it were available?” Response options were 1 (Not at all likely) to 5 (Extremely likely).

Next, we assessed injunctive norms, a construct that measures the extent to which a participant’s peers would approve or disapprove of their behaviors [50]. We measured injunctive norms by querying agreement with the statement, “People who are important to me think I should buy environmentally sustainable foods.” Likewise, we measured descriptive norms, a construct that measures perceptions that other people engage in a given behavior [50]. We measured descriptive norms by querying agreement with the statement, “Most shoppers buy environmentally sustainable foods when they are shopping for groceries.” Response options for both norm questions ranged from 1 (Strongly disagree) to 5 (Strongly agree).

We then assessed acceptability of the climate-impact labels, climate-focused social comparison message, and healthy and sustainable swap recommendations with 1 statement for each intervention component (e.g., “These labels would help me choose more environmentally sustainable foods.”). Responses for all 3 questions ranged from 1 (Not at all) to 5 (A great deal). Participants in both the experimental and control arms were shown the same sample images of the interventions. We also collected the following sociodemographic characteristics: age, gender, race and ethnicity, education, financial situation (using the subjective financial situation measured developed by Williams et al. [51]), employment, political beliefs, red meat consumption, and interest in sustainability (using an adapted scale developed by Haws et al. [52]). All survey measures are reported in Supplemental Table 2.

### Statistical analysis

Study hypotheses and analyses were preregistered prior to data collection at clinicaltrials.gov (NCT06119165) and aspredicted.org (#147,997; https://aspredicted.org/tm8b-tds6.pdf). All analyses were conducted in Stata version 17. We reported this trial according to CONSORT guidelines (**CONSORT Checklist**). Per CONSORT guidelines, we did not test for a balance in covariates [53]. For all statistical tests, we used a 2-sided critical alpha of 0.05.

First, to evaluate the impact of the intervention on the primary and secondary outcomes, we assessed whether the outcomes varied by trial arm using linear regression, regressing the outcome on an indicator variable for trial arm (experimental compared with control). Second, we conducted prespecified exploratory moderation analyses to determine whether the impact of the environmental nudges on healthfulness varied by the following participant characteristics: gender, educational attainment, income, frequency of red meat intake, political orientation, and interest in sustainability. To do this, we fit a series of linear regression models (one for each potential moderator), with trial arm, the moderator, and their interaction as predictors. We also evaluated the impact of the climate-impact label intervention alone (before exposure to the peer comparison message and swap recommendations) on the healthfulness and carbon footprint of initial food selections using linear regression, regressing the outcome on an indicator variable for trial arm (not preregistered). Finally, we described the percentage of participants who were offered and accepted swaps in each food category (not preregistered).

To our knowledge, this was the first study to test the effects of “stealthy” sustainability-focused nudges that also incorporated health considerations on the healthfulness of food selections. Thus, we powered the study to detect a small, standardized effect of Cohen’s *d* = 0.15. The target sample size of 2000 (1000 per arm) provides 90% power to detect a difference in means between the experimental and control arms of *d =* 0.15 or larger, assuming α = 0.05.

**FIGURE 2:**
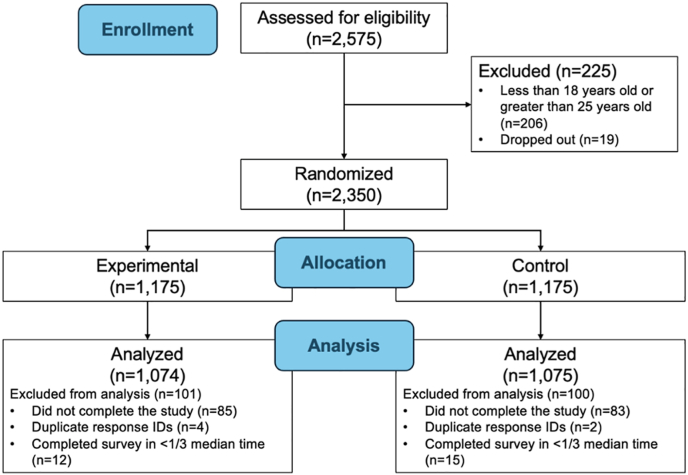
Participant flow chart (*n* = 2149).

We excluded participants from analysis if they dropped out before completing the full survey (preregistered) or had duplicate response IDs (not preregistered). Due to the high prevalence of bots in online research [54], bot prevention measures are important. The survey research firm used, CloudResearch, vets all participants prior to entry into the survey to prevent inattentive respondents, bots, and frauds [55]. In addition, to further screen out bots, we excluded participants who completed the survey in less than one-third the median completion time (preregistered). A total of 2149 participants were included in the final analyzed sample (Figure 2).

## Results

### Descriptives

Participant sociodemographic characteristics are reported in Table 1 [52]. The majority of the sample was Non-Hispanic White (69% in experimental arm and 66% in control arm), ate red meat ≥2 times/wk (59% in experimental arm and 61% in control arm), and had a moderate-high or high interest in sustainability (70% in experimental arm and 68% in control arm). Approximately half of the sample identified as women (51% in experimental arm and 52% in control arm) and held moderate political beliefs (50% in experimental arm and 49% in control arm),

**TABLE 1:** Sociodemographic characteristics of the sample by trial arm (*n* = 2149)

	Experimental arm (*n* = 1074)		Control arm (*n* = 1075)	
	*n*	%	*n*	%
Age, y, mean (SD)	21.5	2.4	21.4	2.3
Gender				
Woman	551	51	559	52
Man	497	46	488	45
Other gender identity	26	2	28	3
Race and ethnicity				
Hispanic, Latino, or Spanish	100	9	112	10
NH White	741	69	711	66
NH Black or African American	145	14	158	15
NH Asian	38	4	29	3
NH other racial identity	15	1	21	2
NH Multiracial	35	3	44	4
Education				
High school diploma or GED or lower	520	48	544	51
Some college	300	28	317	29
Associate’s degree or higher	254	24	214	20
Financial situation				
Does not meet basic expenses	89	9	68	7
Just meets basic expenses	311	30	334	32
Meets needs with a little left	312	30	312	30
Lives comfortably	316	31	317	31
Work situation				
Unemployed	380	35	404	38
Part-time or temporary work	293	27	295	27
Full-time work or >1 job	401	37	376	35
Political beliefs				
Conservative	237	22	273	25
Moderate	537	50	529	49
Liberal	299	28	273	25
Red meat consumption				
Never or <1 time/wk	255	24	218	20
1 time/wk	183	17	193	18
2–3 times/wk	378	35	392	36
4–6 times/wk	153	14	153	14
≥1 times/d	105	10	118	11
Interest in sustainability				
Low	100	9	99	9
Moderate-low	223	21	248	23
Moderate-high	477	44	506	47
High	274	26	222	21

Abbreviations: GED, General Educational Development (high school equivalency diploma); NH, non-Hispanic.

Gender response options included: Woman; Man; Neither woman nor man; Prefer to self-describe; Prefer not to say. Race and ethnicity response options included: Hispanic, Latino, or Spanish; White; Black or African American; Asian; American Indian or Alaska Native; Middle Eastern or Northern African; Native Hawaiian or Other Pacific Islander; Some other race or ethnicity. Interest in sustainability measured using an adapted 3-item GREEN scale [52] (Haws et al., 2014).

Among participants in the experimental arm, 17% initially selected a high climate-impact snack and thus were shown a social comparison message and were offered swaps. Of these participants, 49% accepted the swap. In the protein category, 22% of experimental arm participants initially selected a high climate-impact food and were shown a social comparison message and offered swaps. Of these participants, 56% accepted the swap. In the frozen meal category, 20% of experimental arm participants initially selected a high climate-impact food and were shown a social comparison message and offered swaps. Of these participants, 54% accepted the swap (Supplemental Table 3).

### Primary outcome

**FIGURE 3:**
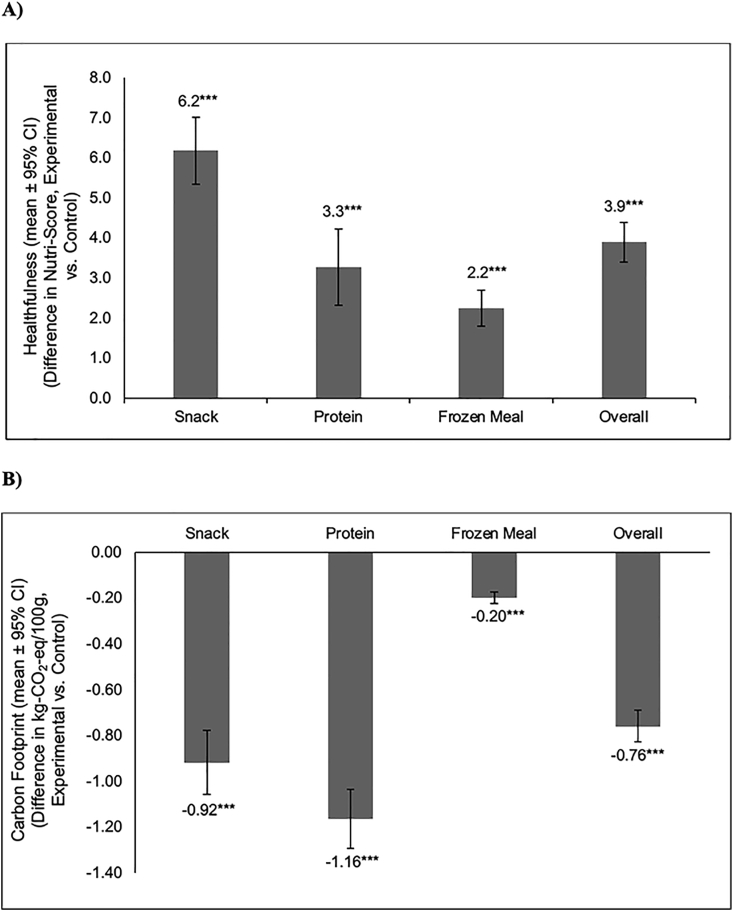
Impact of sustainability-focused nudges about healthfulness (A) and carbon footprint (B) on participants’ selections by product category (*n* = 2149). Difference is experimental minus control. The inverted Nutri-Score scale for products included in this study ranged from −12 to 30, with a higher score indicating a higher nutritional value (i.e., healthier product) and a lower score indicating a lower nutritional value (less healthy product). Carbon footprint is reported in kg CO_2_-eq/100 g, i.e., kilograms of carbon dioxide equivalents per 100 g of food. Carbon footprint data came from the dataFRIENDS database; values for specific products ranged from 0.00 to 5.42 kg CO_2_-eq/100 g [41, 42]. Bars show 95% confidence interval (CI). *P* values calculated using linear regressions. ∗∗∗*P* < 0.001. To calculate the “Overall” Nutri-Score and carbon footprint, we calculated each participant’s mean Nutri-Score and carbon footprint across the 3 food categories, then used this value in the linear regression model.

For all 3 product categories, participants in the experimental arm selected products with higher Nutri-Scores (that is, products with higher nutritional values) compared with participants in the control arm (Figure 3A [41,42]; Supplemental Table 4). In the snack category, participants in the experimental arm selected products that were 6.2 points healthier than participants in the control arm (10.5 compared with 4.3, *P* < 0.001, *d* = 0.63). In the protein category, participants in the experimental arm selected products that were 3.3 points healthier than participants in the control arm (20.4 compared with 17.2, *P* < 0.001, *d* = 0.29). In the frozen meal category, participants in the experimental arm selected products that were 2.3 points healthier than participants in the control arm (20.1 compared with 17.8, *P* < 0.001, *d* = 0.42). When all 3 choices were averaged, participants in the experimental arm selected products that were 3.9 points healthier than participants in the control arm (17.0 compared with 13.1, *P* < 0.001, *d* = 0.67). The impact of the sustainability-focused nudges on healthfulness of participants’ selections was not moderated by gender, education, financial situation, red meat consumption, political beliefs, or interest in sustainability (Table 2; *P* values ranged between 0.13 and 0.99). Furthermore, when examining the impact of the climate-impact labels alone on the healthfulness of food selections (before exposure to the peer comparison messages and swap recommendations), participants in the experimental arm selected products with a higher nutritional value compared with participants in the control arm, although the differences and effect sizes were smaller (Supplemental Table 5; differences ranged between 1.4 and 3.8 points, *d* values ranged between 0.13 and 0.40).

**TABLE 2:** Moderation of the impact of sustainability-focused nudges on healthfulness of food selections by sociodemographic characteristics

	ADE	95% CI	*P*
Gender			
Woman	3.9	(3.3, 4.6)	0.99
Man	3.9	(3.2, 4.7)	
Education			
High school diploma or GED or lower	4.1	(3.4, 4.8)	0.48
Some college	3.4	(2.5, 4.3)	
Associate’s degree or higher	3.9	(2.8, 4.9)	
Financial situation			
Does not meet basic expenses	3.6	(1.7, 5.4)	0.53
Just meets basic expenses	4.3	(3.4, 5.2)	
Meets needs with a little left	3.4	(2.5, 4.3)	
Lives comfortably	3.7	(2.8, 4.6)	
Red meat consumption			
Never or <1 time/wk	2.9	(1.9, 3.9)	0.13
1 time/wk	3.4	(2.2, 4.6)	
2–3 times/wk	4.6	(3.8, 5.4)	
4–6 times/wk	4.2	(2.9, 5.5)	
≥1 times/d	3.7	(2.2, 5.2)	
Political beliefs			
Conservative	4.2	(3.2, 5.2)	0.49
Moderate	3.6	(2.9, 4.3)	
Liberal	4.1	(3.2, 5.1)	
Interest in sustainability			
Low	2.4	(0.8, 3.9)	0.23
Moderate-low	3.9	(2.9, 5.0)	
Moderate-high	3.8	(3.1, 4.5)	
High	4.3	(3.3, 5.4)	

Abbreviations: ADE, average differential effect; CI, confidence interval; GED, General Educational Development (high school equivalency diploma).

*P* values calculated using Wald test. Healthfulness defined as the inverted continuous Nutri-Score score of food selections.

### Secondary outcomes

A similar pattern was observed for the carbon footprint of products selected. For all 3 product categories, participants in the experimental arm selected products with a lower carbon footprint than those the participants in the control arm selected (Figure 3B; Supplemental Table 6). Participants in the experimental arm selected snack products with carbon footprint 0.92 kg CO_2_-eq/100 g lower than the snack products selected by participants in the control arm (0.47 compared with 1.39 kg CO_2_-eq/100 g, *P* < 0.001, *d* = 0.56). Participants in the experimental arm selected protein products with carbon footprint 1.16 kg CO_2_-eq/100 g lower than the protein products selected by participants in the control arm (0.79 compared with 1.95 kg CO_2_-eq/100 g, *P* < 0.001, *d* = 0.76). Likewise, participants in the experimental arm selected frozen meal products with carbon footprint 0.20 kg CO_2_-eq/100 g lower than the snack products selected by participants in the control arm (0.33 compared with 0.52 kg CO_2_-eq/100 g, *P* < 0.001, *d* = 0.67). On average, participants in the experimental arm selected products with an average carbon footprint of 0.53 kg CO_2_-eq/100 g, while participants in the control arm selected products with an average carbon footprint of 1.29 kg CO_2_-eq/100 g (difference = −0.76, *P* < 0.001, *d* = 0.93). Furthermore, when examining the effect of the climate-impact labels alone on the carbon footprint of food selections (prior to exposure to the peer comparison messages and swap recommendations), participants in the experimental arm selected products with a lower carbon footprint compared with participants in the control arm, although the differences and effect sizes were smaller (Supplemental Table 7; differences ranged from −0.13 to −0.70 kg CO_2_-eq/100g, *d* values ranged from 0.37 to 0.43).

Compared with participants in the control arm, participants in the experimental arm reported that they thought more about environmental sustainability (difference = 0.4, *P* < 0.001, *d* = 0.34), less about taste (difference = −0.1, *P* < 0.001, *d* = 0.14) and cost (difference = −0.1, *P* = 0.02, *d* = 0.10), and similar amounts about healthfulness (difference = 0.0, *P* = 0.32, *d* = 0.04) when selecting products. Participants in the experimental arm reported lower descriptive norms for buying sustainable foods than participants in the control arm (difference = −0.1, *P* = 0.04, *d* = 0.09) but did not differ from the control arm on injunctive norms to buy sustainable foods (Table 3).

**TABLE 3:** Impact of sustainability-focused nudges on psychological outcomes (*n* = 2149)

Psychological outcomes	Experimental arm (*n* = 1074)	Control arm (*n* = 1075)	Difference (95% CI)	*P* value	Cohen’s *d*
	Adjusted mean (95% CI)	Adjusted mean (95% CI)			
Cognitive elaboration					
Environmental sustainability	3.0 (2.9, 3.1)	2.6 (2.5, 2.7)	0.4 (0.3, 0.5)	<0.001	0.34
Health	3.3 (3.2, 3.4)	3.2 (3.2, 3.3)	0.0 (0.0, 0.1)	0.32	0.04
Taste	4.2 (4.2, 4.3)	4.4 (4.3, 4.4)	−0.1 (−0.2, −0.1)	<0.001	0.14
Cost	3.4 (3.3, 3.4)	3.5 (3.4, 3.6)	−0.1 (−0.2, 0.0)	0.02	0.10
Perceptions and intentions related to sustainable products					
Perceived sustainability	3.9 (3.8, 3.9)	3.2 (3.1, 3.2)	0.7 (0.6, 0.8)	<0.001	0.64
Perceived healthfulness	3.6 (3.5, 3.6)	3.3 (3.3, 3.4)	0.2 (0.2, 0.3)	<0.001	0.24
Purchase intentions	2.7 (2.6, 2.8)	2.5 (2.4, 2.5)	0.2 (0.1, 0.3)	<0.001	0.18
Perceptions and intentions related to unsustainable products					
Perceived sustainability	1.8 (1.8, 1.9)	2.6 (2.5, 2.6)	−0.7 (−0.8, −0.6)	<0.001	0.63
Perceived healthfulness	2.4 (2.3, 2.5)	2.6 (2.5, 2.6)	−0.2 (−0.3, −0.1)	<0.001	0.15
Purchase intentions	2.6 (2.5, 2.7)	3.0 (2.9, 3.0)	−0.4 (−0.5, −0.3)	<0.001	0.29
Norms					
Injunctive norms	3.0 (2.9, 3.1)	3.0 (2.9, 3.0)	0.1 (0.0, 0.1)	0.28	0.05
Descriptive norms	2.6 (2.6, 2.7)	2.7 (2.7, 2.8)	−0.1 (−0.2, 0.0)	0.04	0.09

Abbreviation: CI, confidence interval.

All outcomes were measured using a 5-point Likert scale. Difference is experimental minus control. *P* values calculated using linear regressions. Sustainable products received a “LOW CLIMATE IMPACT” label, and unsustainable products received a “HIGH CLIMATE IMPACT” label.

Compared with the control arm, participants in the experimental arm reported higher perceived sustainability (difference = 0.7, *P* < 0.001, *d* = 0.64), perceived healthfulness (difference = 0.2, *P* < 0.001, *d* = 0.24), and purchase intentions (difference = 0.2, *P* < 0.001, *d* = 0.18) of sustainable products (Table 3). Furthermore, participants in the experimental arm reported lower perceived sustainability (difference = −0.7, *P* < 0.001, *d* = 0.63), perceived healthfulness (difference = −0.2, *P* < 0.001, *d* = 0.15), and purchase intentions (difference = −0.4, *P* < 0.001, *d* = 0.29) of unsustainable products.

Exposure to the sustainability-focused nudges led to increased acceptability of both the climate-impact labels (difference = 0.1, *P* = 0.01, *d* = 0.11) and the climate-focused social comparison messages (difference = 0.2, *P* < 0.001, *d*=0.17) compared with the control arm, but not the healthy and sustainable swap recommendations (difference = 0.1, *P* = 0.31, *d* = 0.04, Supplemental Table 8).

## Discussion

In this online randomized experiment of United States young adults, exposure to sustainability-focused nudges with a hidden health dimension (climate-impact labels, climate-focused social comparison messages, and healthy and sustainable swap recommendations) led to healthier food selections compared with the control arm; the 3.9-point difference in the continuous Nutri-Score score is equivalent to a 25.9% improvement in healthfulness of the selections. Exposure to these sustainability-focused nudges also led to lower carbon footprint of selected foods compared with the control arm; the average difference of −0.76 kg CO_2_-eq/100 g is equivalent to ∼1.9 fewer miles driven by a gasoline-powered vehicle [56]. This trend was observed both at the food category level and when selections were averaged. Healthfulness scores were low across the study population. Moreover, improvements in healthfulness and carbon footprints were modest in magnitude. Additional interventions may be needed to further improve diets and carbon footprints. Nevertheless, these are low-cost, highly scalable interventions that interventionists could use to “stealthily” improve dietary quality while appealing only to sustainability values. These results suggest that sustainability-focused nudges are a promising method for improving both the healthfulness and sustainability of United States young adults’ diets when they are designed to also incorporate healthfulness considerations (e.g., only offer recommendations to sustainable foods that are also healthy).

To our knowledge, this is one of the first studies to test the effect of “stealthy” sustainability-focused nudges with a hidden health dimension on the healthfulness of food selections. Similar to our results, an online experiment conducted by Wolfson et al. [57] found that exposure to low or high climate-impact labels was associated with more sustainable food choices, although only the high climate-impact labels were associated with more healthful food choices. Notably, Wolfson and colleagues examined fast-food offerings, none of which were classified as “healthy,” whereas our study explicitly offered recommendations only for sustainable foods that were also healthy. This difference in food offerings may explain the different effects on healthfulness between the 2 studies. Alternatively, differences between the studies might be because participants in the prior study saw only low climate-impact or only high climate-impact labels, whereas participants in our study saw both.

In addition to improvements in healthfulness, the nudges also improved the environmental sustainability of participants’ selections (i.e., reduced their carbon footprint). A prior study conducted in an Italian workplace canteen used an intervention combining health and environmental menu labeling with a web application that suggested meals for purchase in the canteen and provided feedback on the nutritional and environmental impacts of employees’ purchases. These interventions were associated with healthier food choices, but only minimal improvements were seen on the environmental impact of food choices [58]. Our study’s larger effects on sustainability could be attributed to the combination of nudges used in our study, particularly the use of swaps, as a 2021 systematic review of found that interventions that offer alternatives were successful at encouraging adoption of sustainable food choices [59].

The nudge interventions worked to improve healthfulness and sustainability of food selections across product categories, including for snacks, proteins, and frozen meals. These results align with a prior experiment testing the impact of a dish placement and labeling intervention, which similarly found that those nudges led to increased selection of healthy and sustainable dishes across each dish category [60]. The nudge interventions lead to the smallest improvements in healthfulness and sustainability in the frozen meal category, despite participants being offered and accepting swaps at a similar rate in this category as in the snack and protein categories. The smaller improvements in this category likely reflect the narrower range of options in terms of healthfulness and carbon footprints in this category. Additionally, the frozen meals offered to participants included multiple ingredients across several food groups (i.e., meats, grains, dairy, and vegetables), whereas most foods offered within the snack and protein categories contained ingredients from a single food group. Indeed, the health and ecological impact of a meal depends on the procurement and preparation of all components [61], so the extra steps required for processing the multiple ingredients in a frozen meal may explain the narrower range of options in terms of healthfulness and carbon footprints [62]. Nevertheless, even in the frozen meal category, the nudge interventions improved healthfulness by 11.9% and reduced carbon footprint by 46.6%.

Exposure to sustainability-focused nudges led to increased thinking about the food’s sustainability when making selections, although there were no differences in participants’ thinking about the healthfulness of foods when making selections. Evidence from randomized trials testing the effect of cigarette and sugary drink warning labels indicates that increased cognitive elaboration (i.e., thinking about the harms of cigarettes and sugary drinks) can predict behavior change [63,64]. The increased thinking about sustainability may explain the increased selection of sustainable foods among study participants exposed to the nudges. Furthermore, when reporting perceptions and intentions related to individual foods, those who were exposed to the nudges reported higher perceived sustainability and perceived healthfulness of sustainable foods and lower perceived sustainability and perceived healthfulness of unsustainable products compared with participants in the control arm. These results suggest that young adults may have existing notions that sustainable foods are also more healthful. These perceptions are generally warranted, given that evidence suggests that a sustainable diet is also more healthful [16,17].

The effects of the nudge interventions did not differ by gender, education, financial situation, red meat consumption, political beliefs, or interest in sustainability. We were surprised to observe no moderation by red meat consumption or interest in sustainability, as prior research indicates that individuals with lower red meat consumption and higher interest in sustainability are more likely to engage in sustainable dietary behaviors [65,66]. However, previous climate labeling online experiments similarly found no moderation by red meat consumption [67] or climate change beliefs [57,68]. The observed lack of moderation suggests that the combination of climate-impact labels, climate-focused social comparison messages, and healthy and sustainable swap recommendations may be successful at improving the healthfulness of food selections for a wide range of young adults in the United States. Future research should assess whether similar patterns of moderation are observed for these nudge interventions in real-world purchasing settings.

This study has several public health implications. The majority of study participants found the intervention components acceptable, suggesting that these nudges would be received positively by United States young adults. For the climate-impact labels, this is in line with prior evidence that indicates that consumers are supportive of sustainability labeling [69]. In the United States, some food corporations and restaurants have introduced voluntary sustainability labeling schemes; for example, Sweetgreen [70] and Just Salad [71] display GHGE estimates alongside each menu item. These early corporate initiatives, combined with our study results, underscore the policy relevance of research assessing the effects of climate-impact labels on healthfulness and sustainability. The other nudges tested, social comparison messages and swap recommendations, would be most feasible in an online environment, such as online grocery shopping platforms or restaurant delivery apps. Future research should test this combination of interventions in real-world settings to further understand the applicability of these interventions.

Strengths of this study include the randomized design and the preregistration of study analyses and hypotheses on clinicaltrials.gov and AsPredicted.org. Furthermore, we assessed healthfulness and sustainability using validated measures [41,42,72]. Limitations include the use of a convenience sample, the short-term exposure to the nudges in the context of an online experiment, and the fact that participants did not see food prices nor have to pay for food selections. Additionally, this experiment measured hypothetical food selections. Although the Theory of Reasoned Action and Theory of Planned Behavior suggest that intentions predict behavior [73,74], hypothetical selections may not accurately reflect real-world food purchasing and consumption behaviors [75,76]. These nudges should be tested with probability samples, in real-world stores, and using longitudinal designs that capture consumption behavior. Moreover, GHGE were estimated using a database of similar products [42] rather than calculated using exact recipe ingredients and quantities, which may limit the accuracy of these values. Finally, we implemented guardrails on the nutritional profiles of the swaps, preventing participants from swapping to less healthful foods with lower levels of carbon pollution, which may have amplified the effect seen on the healthfulness of food selections.

In conclusion, this online randomized experiment of 2149 young adults (aged 18–25 y) in the United States tested the impact of a suite of “stealthy” sustainability-focused nudges (climate-impact labels, social comparison messages, and healthy and sustainable swap recommendations) on the healthfulness of food selections. Compared with the no-intervention control arm, participants exposed to the nudge interventions selected foods that were more healthful and more sustainable. Exposure to the nudges also led to increased thinking about the sustainability of foods when making selections, indicating that sustainability-focused nudges may help drive behavior change through increased awareness of a food’s climate impact. These results suggest that “stealthy” sustainability-focused nudges may be an effective tool to help shift United States young adults’ dietary behaviors toward more healthful and sustainable foods if they are designed to incorporate healthfulness (e.g., only recommend sustainable foods that are also healthy). Future research should evaluate real-world effects of this suite of interventions.

## Author contributions

The authors’ responsibilities were as follows – AHG, LST: conceptualization; CEP: formal analysis; CEP, AHG, SMF, CD, LST: investigation; CEP, AHG, SMF, LST: methodology; CEP: project administration; AHG, LST: supervision; LST: funding acquisition; CEP, AHG, LST: writing – original draft; SMF, CD: writing – review & editing; and all authors: read and approval the final manuscript.

## Data availability

The datasets and code generated during the current study are available in the UNC Dataverse repository, https://doi.org/10.15139/S3/Q9IFNB.

## Declaration of Generative AI and AI-assisted Technologies in the Writing Process

The authors declare that no generative AI or AI-assisted technologies were used in the writing of this manuscript.

## Funding

This work was funded through a grant from the Wellcome Trust, grant ID #216042/Z/19/Z. CEP received support from the Population Research Training grant (T32 HD007168) and the Population Research Infrastructure Program (P2C HD050924) awarded to the Carolina Population Center at the University of North Carolina at Chapel Hill by the Eunice Kennedy Shriver National Institute of Child Health and Human Development. K01 HL158608 from the National Heart, Lung, and Blood Institute of the NIH supported AHG’s time. The funders had no role in the study design; collection, analysis and interpretation of data; or writing of the report.

## Conflict of interest

The authors report no conflicts of interest.
