# Supplementary material for: Nudging Toward Sustainable and Healthy Diets: A Randomized Trial of Young Adults
Source: Curr Dev Nutr. 2026 Jun 1;10(7):109384. doi: 10.1016/j.cdnut.2026.109384 (PMC13315694; doi:10.1016/j.cdnut.2026.109384)
Supplement: Multimedia component 1 [file mmc1.docx]

**Supplementary Material**

**Prestemon C.E., et al. Nudging towards sustainable and healthy diets: A randomized trial of young adults.**

**Supplemental Methods**

***Nutri-Score***

Two trained research assistants entered information on the nutrient content of products (energy; sugars; saturated fats; salt; protein; fiber; fruits, vegetables, and legumes) per 100g. Energy was converted from kilocalories to kilojoules using the standard formula of 1 kilocalorie = 4.184 kilojoules. Sodium was converted to salt using the formula: salt = sodium x 2.5 (1). Scores were then assigned for unfavorable components [energy (kJ), sugars (g), saturated fats (g), and salt (g)] and for favorable components [proteins (g), fiber (g), and fruits, vegetables, legumes (% weight)]. Compared to the original Nutri-Score algorithm (2), the most notable difference in the 2022 Nutri-Score update is that the protein component could only be scored a maximum of two points for products containing red or processed meat, with the rationale to reflect the health risks associated with high intake of these foods (3, 4). A third researcher checked the entered product information and component scores. All three met to resolve any discrepancies.

Products were classified into one of three categories in accordance with the Nutri-Score algorithm: general foods; meat; or fats, oils, nuts, and seeds. Once the nutrition information and component scores were entered, the Nutri-Score algorithm for the product category (general foods; meat; fats, oils, nuts, and seeds) was applied as described in the Nutri-Score report (1). For all foods, the two research assistants again independently calculated the algorithm, the third researcher checked the calculations, and all three met to resolve discrepancies. For the purposes of interpretability, we inverted the Nutri-Score scale after scoring. The inverted Nutri-Score scale (for foods included in this study) ranged from -12 to 30, with a lower score indicating a lower nutritional value and a higher score indicating a higher nutritional value.

To further ensure accuracy of score calculations, the procedures for calculating the Nutri-Score were independently recreated and approved by a registered dietitian. Two additional study team members who were not involved in the original scoring process also recalculated the Nutri-Scores for a random subset of the products.

***Carbon footprint***

Two trained research assistants matched each product to its closest NHANES food. For example, in the frozen food category, the food “Marie Callender’s Roasted Garlic Chicken Bowl” was matched with the NHANES Food “Chicken in cream sauce with noodles and vegetable, frozen meal”; in the protein category, the food “Fresh Atlantic Salmon Portions” was matched with the NHANES food “Salmon, raw”; and in the Snack category, the food “Double Stuf Oreos” was matched with the NHANES food “Cookie, chocolate, sandwich, with extra filling.” A third researcher checked the product matchings. All three met to resolve any discrepancies. To ensure accuracy of product matching, two additional study team members reviewed a random subset of the products. Within product subcategories, products were divided into tertiles (low-, medium-, and high-carbon footprint). The entire study team met and approved these tertile assignments.

| **Table S1.** Median and interquartile range of Nutri-Score and greenhouse gas emissions of foods included in selection task. | | |
| --- | --- | --- |
|  | **Nutri-Score** | **kg CO_2_-eq/100g** |
|  | **Median (IQR)** | **Median (IQR)** |
| Snack | 7 (4-18) | 0.17 (0.09-0.65) |
| Protein | 24 (14-27) | 0.51 (0.37-3.62) |
| Frozen | 23 (20-24) | 0.33 (0.17-0.62) |
| All foods | 21 (12-25) | 0.37 (0.16-0.79) |

**Table S2.** Codebook.

| **Construct**  **Question ID**  **(source)** | **Measure** | **Response options (where applicable)** |
| --- | --- | --- |
|  | **SCREENER** |  |
| **scr_intro** | Welcome! This survey will ask you questions about your experience with purchasing and eating food.  [Page Break] |  |
| **age** | How old are you? Enter your age in years. | Open ended  [Add skip logic – if under 18 y/o or older than 25 y/o skip to end, restrict 0-99] |
| **gender** | Select the option that best describes your gender. | 1 = Woman  2 = Man  3 = Neither woman nor man  4 = Prefer to self-describe: _______  5 = Prefer not to say |
| **race and ethnicity (5)** | What is your race and/or ethnicity? Select all that apply. | 1 = White  2 = Hispanic, Latino, or Spanish  3 = Black or African American  4 = Asian  5 = American Indian or Alaska Native  6 = Middle Eastern or Northern African  7 = Native Hawaiian or Other Pacific Islander  8 = Some other race or ethnicity (please specify): _____ |
| **education** | What is the highest level of school you have completed? | 1 =Less than high school or U.S. high school equivalent (GED)  2 =High school diploma or U.S. high school equivalent (GED)  3 =Some college  4 =Associate’s degree  5 =4-year college degree  6 =Graduate degree |
| **college** | Are you currently enrolled **full-time** at a college or university? | 1 = Yes  0 = No  [quota set to at least 25% “yes”] |
| **EXPERIMENTAL ARM** | | |
| Prompt | Please imagine you are shopping online for groceries for yourself. We will show you three food categories and ask you to select one food from each category that you would like to purchase. You will not be required to pay for these items, and you will not receive them, but please shop as you normally would.  [page break] |  |
| Prompt | The foods you see will carry a label that shows the climate impact from producing these foods. These climate labels look like this:  **[insert image of climate labels]**  Green labels mean the food has low climate impact. Producing these foods creates low carbon pollution (or in other words, causes little damage to the environment).  Yellow labels mean the food has medium climate impact. Producing these foods creates medium carbon pollution (or in other words, causes medium damage to the environment).  Red labels mean the food has high climate impact. Producing these foods creates high carbon pollution (or in other words, causes high damage to the environment).  You will see these labels on foods in the next section.  **[page break]** |  |
| Programming notes | **[randomize participants to view protein, frozen meal, and snack questions in random order]** |  |
| Protein selection  proteinchoice_eco | Please select 1 food from this category that you would like to purchase.  **[Display products for this category with ecolabels, shown in random arrangement]**  **[page break]** | 1=Great Value Frozen Peeled Tail on Extra Large Shrimp, 12 oz (Frozen)  2=Beef Choice Angus Ribeye Steak, 1.5-2.6lb, Tray  3=Ground Beef Burgers, 93% Lean/7%Fat, 4ct, 1lb, Tray (Fresh)  4= Ground Beef Chuck 80% Lean/20% Fat, 1lb, Tray (Fresh)  5= Ball Park Bun Length Beef Hot Dogs, 8ct, 15oz  6= Pork Center Cut Loin Chops Boneless Family Pack, 2.0-3.8 lbs, Tray (Fresh)  7= Great Value Hickory Smoked Bacon,12 oz  8=Johnsonville Jalapeno & Cheddar Smoked Sausage, 6ct, 14oz  9=Tyson All Natural Boneless Skinless Chicken Breasts, 1.75 - 3.0 lb Tray (Fresh)  10=Fresh Atlantic Salmon Portions  11= Great Value Wild Caught Pacific Cod Filets, 2lb (Frozen)  12=Jennie-O 93% Lean Turkey, 16oz tray  13= Tyson fully cooked and breaded chicken patties, 1.62lb (Frozen)  14= Butterball Original Seasoned Frozen Turkey Burgers, 6ct, 2lb  15= Beyond Burger Plant-Based Patties  16=Morning Star Farms Grillers Original  17=Gardein The Ultimate Beefless Ground, 13.7 oz Bag  **[force response]**  **[red label options: 1-5]** |
| Protein feedback | **[display if participant selected a protein with a red eco-label]**  [display social comparison report image] |  |
| Protein swap  protein_swap | **[display if participant selected a protein with a red eco-label (proteinchoice_eco = 1-5)]**  **[Show image of swap product]**  The food you selected receives a red climate label, meaning this food has high climate impact. The following foods would reduce your climate impact. Would you like to switch to one of these foods?  Select the food you would like to switch to, or select “I don’t want to switch”.  **[page break]** | 9=Tyson All Natural Boneless Skinless Chicken Breasts, 1.75 – 3.0 lb Tray (fresh)  10=Fresh Atlantic Salmon Portions  14=Butterball Original Seasoned Frozen Turkey Burgers, 6 ct, 2lb  16=Morning Star Farms Grillers Original  0=I don’t want to switch  **[force response]** |
| Frozen meal selection  frozenchoice_eco | Please select 1 food from this category that you would like to purchase.  **[Display products for this category with ecolabels, shown in random arrangement]**  **[page break]** | 1=Marie Callendar's Beef Pot Roast Bowl  2= El Monterey XX Large Beef and Bean Green Chili Burrito  3=Hungry-Man Salisbury Steak  4=Tostino’s Original Crisp Crust Pizza Three Meat Frozen Pizza 10.5 oz  5=Marie Callendar's Beef & Broccoli Bowl Frozen Meal  6=Healthy Choice Café Steamers Beef Teriyaki Frozen Meal  7= Red Baron Frozen Pizza Deep Dish Singles Pepperoni 2 pack  8= Banquet Mega Bowls Dynamite Penne & Meatballs  9= Healthy Choice Simply Steamers Grilled Chicken Marsala Frozen Meal  10= El Monterey XX Large Bean and Cheese Burrito  11= Marie Callender's Sweet & Sour Chicken  12= Marie Callender's Roasted Garlic Chicken Bowl  13= Healthy Choice Simply Steamers Chicken & Vegetable Stir Fry  14= Stouffer's Baked Chicken Frozen Meal  15=Healthy Choice Steams Cream Spinach and Tomato Linguini  16=Hungry-Man Roasted Carved Turkey Breast Frozen Dinner  17=Healthy Choice Simply Steamers Unwrapped Burrito Bowl  **[force response]**  **[red labels: choices 1-6]** |
| Frozen meal feedback | **[display if participant selected a frozen meal with a red eco-label (frozenchoice_eco=1-6)]**  [display social comparison report image] |  |
| Frozen meal swap  frozen_swap | **[display if participant selected a frozen with a red eco-label]**  **[Show image of swap product]**  The food you selected receives a red climate label, meaning this food has high climate impact. The following foods would reduce your climate impact. Would you like to switch to one of these foods?  Select the food you would like to switch to, or select “I don’t want to switch.”  **[page break]** | 9=Healthy Choice Simply Steamers Grilled Chicken Marsala Frozen Meal  12=Marie Callender’s Roasted Garlic Chicken Bowl  15=Healthy Choice Steams Cream Spinach and Tomato Linguini  17=Healthy Choice Simply Steamers Unwrapped Burrito Bowl  0=I don’t want to switch  **[force response]** |
| Snack selection  snackchoice_eco | Please select 1 food from this category that you would like to purchase.  **[Display products for this category with ecolabels, shown in random arrangement]**  **[page break]** | 1=Jack Links Beef Jerky Teriyaki  2= Slim Jim Original Smoked Snack Sticks 26 count  3= Frigo Cheese Heads Original Mozzarella String Cheese, 16 Oz, 16 Ct  4= P3 Turkey, Ham & Cheddar Cheese Protein Snack Pack Pieces, 2.3 oz Tray  5= Great Value Cheese Dip & Breadsticks Snacks, 1 oz, 5 Count  6= NUT-rition Heart  7= Planters Lightly Salted Mixed Nuts  8=Double Stuf Oreos  9=Great Value Dried Apricots  10=Ruffles Party Size Original  11=Newtons Soft & Chewy Fig Cookie  12=Harvest Snaps Lightly Salted Baked Green Pea Snacks, 6 oz  13=Bada Bean Bada Boom Sea Salt Crunchy Broad Beans, 4.5 oz  14=Doritos Nacho Cheese, Party Size  15=Teddy Grahams Honey Graham Snacks  16=Skinny Pop Popcorn Original  17=Snyder’s of Hanover Unsalted Pretzels, 12 oz  **[force response]** |
| Snack feedback | **[display if participant selected a snack with a red eco-label]**  [display social comparison report image] |  |
| Snack swap  snack_swap | **[display if participant selected a snack with a red eco-label (snackchoice_eco=1-5)]**  **[Show image of swap product]**  The food you selected receives a red climate label, meaning it has high climate impact. The following foods would reduce your climate impact. Would you like to switch to one of these foods?  Select the food you would like to switch to, or select “I don’t want to switch”.  **[page break]** | 17= Snyder’s of Hanover Unsalted Pretzels, 12 oz  12=Harvest Snaps Lightly Salted Baked Green Pea Snacks, 6 oz  13=Bada Bean Bada Boom Sea salt Crunch Broad Beans, 4.5 oz  0=I don’t want to switch  **[force response]**  **[randomize order of products]** |
| **CONTROL ARM** | | |
| Prompt | Please imagine you are shopping online for groceries for yourself. We will show you three food categories and ask you to select one food from each category that you would like to purchase. You will not be required to pay for these items, and you will not receive them, but please shop as you normally would.  **[page break]** |  |
| Programming notes | **[randomize participants to view protein, frozen meal, and snack questions in random order]** |  |
| Protein selection  proteinchoice_ctrl | Please select 1 food from this category that you would like to purchase.  **[Display products for this category without eco-labels, shown in random arrangement]**  **[page break]** | 1=Great Value Frozen Peeled Tail on Extra Large Shrimp, 12 oz (Frozen)  2=Beef Choice Angus Ribeye Steak, 1.5-2.6lb, Tray  3=Ground Beef Burgers, 93% Lean/7%Fat, 4ct, 1lb, Tray (Fresh)  4= Ground Beef Chuck 80% Lean/20% Fat, 1lb, Tray (Fresh)  5= Ball Park Bun Length Beef Hot Dogs, 8ct, 15oz  6= Pork Center Cut Loin Chops Boneless Family Pack, 2.0-3.8 lbs, Tray (Fresh)  7= Great Value Hickory Smoked Bacon,12 oz  8=Johnsonville Jalapeno & Cheddar Smoked Sausage, 6ct, 14oz  9=Tyson All Natural Boneless Skinless Chicken Breasts, 1.75 - 3.0 lb Tray (Fresh)  10=Fresh Atlantic Salmon Portions  11= Great Value Wild Caught Pacific Cod Filets, 2lb (Frozen)  12=Jennie-O 93% Lean Turkey, 16oz tray  13= Tyson fully cooked and breaded chicken patties, 1.62lb (Frozen)  14= Butterball Original Seasoned Frozen Turkey Burgers, 6ct, 2lb  15= Beyond Burger Plant-Based Patties  16=Morning Star Farms Grillers Original  17=Gardein The Ultimate Beefless Ground, 13.7 oz Bag  **[force response]** |
| Frozen meal selection  frozenchoice_ctrl | Please select 1 food from this category that you would like to purchase.  **[Display products for this category without eco-labels, shown in random arrangement]**  **[page break]** | 1=Marie Callendar's Beef Pot Roast Bowl  2= El Monterey XX Large Beef and Bean Green Chili Burrito  3=Hungry-Man Salisbury Steak  4=Tostino’s Original Crisp Crust Pizza Three Meat Frozen Pizza 10.5 oz  5=Marie Callendar's Beef & Broccoli Bowl Frozen Meal  6=Healthy Choice Café Steamers Beef Teriyaki Frozen Meal  7= Red Baron Frozen Pizza Deep Dish Singles Pepperoni 2 pack  8= Banquet Meta Bowls Dynamite Penne & Meatballs  9= Healthy Choice Simply Steamers Grilled Chicken Marsala Frozen Meal  10= El Monterey XX Large Bean and Cheese Burrito  11= Marie Callender's Sweet & Sour Chicken  12= Marie Callender's Roasted Garlic Chicken Bowl  13= Healthy Choice Simply Steamers Chicken & Vegetable Stir Fry  14= Stouffer's Baked Chicken Frozen Meal  15=Healthy Choice Steams Cream Spinach and Tomato Linguini  16=Hungry-Man Roasted Carved Turkey Breast Frozen Dinner  17=Healthy Choice Simply Steamers Unwrapped Burrito Bowl  **[force response]** |
| Snack selection  snackchoice_ctrl | Please select 1 food from this category that you would like to purchase.  **[Display products for this category without eco-labels, shown in random arrangement]**  **[page break]** | 1=Jack Links Beef Jerky Teriyaki  2= Slim Jim Original Smoked Snack Sticks 26 count  3= Frigo Cheese Heads Original Mozzarella String Cheese, 16 Oz, 16 Ct  4= P3 Turkey, Ham & Cheddar Cheese Protein Snack Pack Pieces, 2.3 oz Tray  5= Great Value Cheese Dip & Breadsticks Snacks, 1 oz, 5 Count  6= NUT-rition Heart  7= Planters Lightly Salted Mixed Nuts  8=Double Stuf Oreos  9=Great Value Dried Apricots  10=Ruffles Party Size Original  11=Newtons Soft & Chewy Fig Cookie  12=Harvest Snaps Lightly Salted Baked Green Pea Snacks, 6 oz  13=Bada Bean Bada Boom Sea Salt Crunchy Broad Beans, 4.5 oz  14=Doritos Nacho Cheese, Party Size  15=Teddy Grahams Honey Graham Snacks  16=Skinny Pop Popcorn Original  17= Snyder’s of Hanover Unsalted Pretzels, 12 oz  **[force response]** |
| **FOLLOW-UP** | | |
| Prompt | Now we will ask you about the choices you made while you were shopping. |  |
| Noticing ecolabels  noticed | When you were shopping, did you notice climate labels on the foods?  **[page break]** | 1=Yes  0=No |
| Elaboration-stem | We are interested in how you selected foods. When you selected foods, how much did you think about each of the following characteristics?  **[format items below as a matrix; randomize order of attributes]** | 1=Not at all  2=Very little  3=Somewhat  4=Quite a bit  5=A great deal |
| Elaboration-Health  elab_health | Healthfulness |  |
| Elaboration-Taste  elab_taste | Taste |  |
| Elaboration-Cost  elab_cost | Cost |  |
| Elaboration-Environmental sustainability  elab_envr | Environmental sustainability |  |
| **PRODUCT PERCEPTIONS AND INTENTIONS**  **[In Qualtrics: repeat entire block x 2: randomize to display one product category (protein, frozen meal, snacks) for each of 2 treatment arms (control, treatment) and for each 2 product types (sustainable vs. unsustainable). Display appropriate block for each participant’s condition using survey flow]**  **[display these blocks in random order using survey flow]** | | |
| Perceptions Prompt | The next questions are about this food.  **[insert image of item with label from participant’s condition]**  **[protein: ribeye steak (red) and Morningstar Grillers (green)]**  **[snacks: beef jerky (red) and Bada bean (green)]**  **[frozen meals: beef burrito (red) and spinach and tomato linguini (green)]** |  |
| Perceived sustainability | How environmentally sustainable do you think this food is? | 1=Not at all  2=A little  3=Somewhat  4=Very  5=Extremely |
| Perceived healthfulness | How healthy do you think this food is? | 1=Not at all  2=A little  3=Somewhat  4=Very  5=Extremely |
| Purchase intentions | How likely would you be to buy this food in the next month, if it were available? | 1=Not at all likely  2=A little likely  3=Somewhat likely  4=Very likely  5=Extremely likely |
| **NORMS** | | |
| Prompt  Inj_desc_norm | **Say how much you agree or disagree with the next statements.**  **[Format as matrix]** |  |
| Injunctive norms (6)  inj_should | People who are important to me think I should buy environmentally sustainable foods. | 1 = Strongly disagree  2 = Somewhat disagree  3 = Neither agree nor disagree  4 = Somewhat agree  5 = Strongly agree |
| Descriptive norms  desc_believe | Most shoppers buy environmentally sustainable foods when they are shopping for groceries. | 1 = Strongly disagree  2 = Somewhat disagree  3 = Neither agree nor disagree  4 = Somewhat agree  5 = Strongly agree |
| **ACCEPTABILITY** | | |
| Prompt - Labels  Text adapted from Eco-Score website (7) | Some food companies place climate labels on the foods they sell, like the labels below. Green labels mean the food has low climate impact (or in other words, causes little damage to the environment), yellow labels mean the food has medium climate impact (or causes medium damage to the environment), and red labels mean the food has high climate impact (or causes high damage to the environment). You might have seen these labels earlier in the survey.  Look closely at the labels. Then, answer the question below.  **[Insert image of climate labels]** |  |
| Acceptability – help (8)  ecoaccept_help | These labels would help me choose more environmentally sustainable foods.  [page break] | 1=Not at all  2=A little  3=Somewhat  4=Quite a bit  5=A great deal |
| Prompt – Social comparison | Grocery stores could provide shoppers with feedback about the climate impact of their food choices compared to sustainable peer shoppers, like in the image below. You might have seen this type of feedback earlier in the survey.  Look closely at the information. Then, answer the question below.  **[Insert sample image of social comparison]** |  |
| Social comparison acceptability – help (8)  socialaccept_help1 | This type of feedback would help me choose more environmentally sustainable foods.  [page break] | 1=Not at all  2=A little  3=Somewhat  4=Quite a bit  5=A great deal |
| Prompt – Swaps | Grocery stores could provide shoppers who select foods with high climate impact with a recommendation for similar food that has a lower climate impact, like in the image below. You might have seen this type of recommendation earlier in the survey.  Look closely at this recommendation. Then, answer the question below.  **[Insert image of swap as it was shown earlier in the survey]** |  |
| Social comparison acceptability – help (8)  socialaccept_help2 | This type of recommendation for foods with lower climate impact would help me choose more environmentally sustainable foods. | 1=Not at all  2=A little  3=Somewhat  4=Quite a bit  5=A great deal |
| **DEMOGRAPHICS** | | |
| dem_intro | We are asking the next questions to better understand who took our survey.  [Page Break] |  |
| finsit (9) | Considering your own income and the income from any other people who help you, how would you describe your overall financial situation? | 4 = Live comfortably  3 = Meet needs with a little left  2 = Just meet basic expenses  1 = Don’t meet basic expenses |
| work | What is your current work situation? | 1 = Unemployed  2 = Unemployed but not seeking work (e.g., student, retired, disabled, unpaid primary caregiver)  3 = Part-time or temporary work  4 = Full-time work  5 = Two or more part-time or full-time jobs |
| politics (10) | In general, how would you describe your political views? | 1 = Very conservative  2 = Conservative  3 = Moderate  4 = Liberal  5 = Very liberal |
| redmeat (11) | In the past 30 days, how often did you eat red meat?    Red meat includes beef, lamb, pork, sausage, and ham. It also includes processed red meats such as bacon, hot dogs, and lunch meats, and it includes products that contain red meat (for example, canned beef soup).  It **DOES NOT** include chicken, turkey, or seafood products.  [show image of red meats]  [page break] | 0=Never  1=Less than 1 time per week  2=1 time per week  3=2-3 times per week  4=4-6 times per week  5=1 time per day  6=2 times per day  7=3 or more times per day |
| Green scale prompt | **Say how much you agree or disagree with the statements below.**  [randomize order of GREEN scale statements] |  |
| green_noharm (12) | It is important to me that the products I use do not harm the environment. | 1 = Strongly disagree  2 = Somewhat disagree  3 = Neither agree nor disagree  4 = Somewhat agree  5 = Strongly agree |
| green_impact (12) | I consider the potential environmental impact of my actions when making many of my decisions. | 1 = Strongly disagree  2 = Somewhat disagree  3 = Neither agree nor disagree  4 = Somewhat agree  5 = Strongly agree |
| green_habits (12) | My purchase habits are affected by my concern for the environment.  [page break] | 1 = Strongly disagree  2 = Somewhat disagree  3 = Neither agree nor disagree  4 = Somewhat agree  5 = Strongly agree |
| Closure | Anything else you want to tell us? | [Free response] |

| **Table S3.** Swap statistics. | | | | |
| --- | --- | --- | --- | --- |
|  | **Offered swap** | | **Accepted swap** | |
|  | **n** | **%** | **n** | **%** |
| Snack | 184 | 17 | 90 | 49 |
| Protein | 234 | 22 | 132 | 56 |
| Frozen | 219 | 20 | 118 | 54 |
| *Notes.* Percentage offered swap is count of participants offered swap/count of participants in the experimental arm (n=1,074). Percentage accepted swap is count of participants who accepted the swap/count of participants offered swap. | | | | |

| **Table S4.** Mean Nutri-Score and differences by trial arm (n=2,149). | | | | | |
| --- | --- | --- | --- | --- | --- |
|  | **Experimental Arm**  **(n=1,074)** | **Control Arm**  **(n=1,075)** | **Difference (95% CI)** | **p-value** | **Cohen’s d** |
| **Nutri-Score** | **Adjusted Mean (95% CI)** | **Adjusted Mean (95% CI)** |  |  |  |
| Snack | 10.5 (9.9, 11.1) | 4.3 (3.7, 4.9) | 6.2 (5.3, 7.0) | <0.001 | 0.63 |
| Protein | 20.4 (19.8, 21.1) | 17.2 (16.5, 17.8) | 3.3 (2.3, 4.2) | <0.001 | 0.29 |
| Frozen | 20.1 (19.8, 20.4) | 17.8 (17.5, 18.2) | 2.3 (1.8, 2.7) | <0.001 | 0.42 |
| **Overall** | 17.0 (16.7, 17.3) | 13.1 (12.8, 13.4) | 3.9 (3.4, 4.4) | <0.001 | 0.67 |
| *Notes.* The inverted Nutri-Score scale (for foods included in this study) ranged from -12 to 30, with a lower score indicating a lower nutritional value and a higher score indicating a higher nutritional value. CI = confidence interval. Difference is experimental minus control. P-values calculated using linear regressions. To calculate the “Overall” Nutri-Score, we calculated each participant’s mean Nutri-Score across the three food categories, then used this value in the linear regression model. | | | | | |

| **Table S5.** Mean Nutri-Score and differences prior to the peer comparison messages and swap recommendations by trial arm (n=2,149). | | | | | |
| --- | --- | --- | --- | --- | --- |
|  | **Experimental Arm**  **(n=1,074)** | **Control Arm**  **(n=1,075)** | **Difference (95% CI)** | **p-value** | **Cohen’s d** |
| **Nutri-Score** | **Adjusted Mean (95% CI)** | **Adjusted Mean (95% CI)** |  |  |  |
| Snack | 8.1 (7.5, 8.6) | 4.3 (3.7, 4.9) | 3.8 (3.0, 4.6) | <0.001 | 0.40 |
| Protein | 18.6 (18.0, 19.3) | 17.2 (16.5, 17.8) | 1.5 (0.5, 2.4) | 0.003 | 0.13 |
| Frozen | 19.2 (18.9, 19.5) | 17.8 (17.5, 18.2) | 1.4 (0.9, 1.8) | <0.001 | 0.26 |
| **Overall** | 15.3 (15.0, 15.6) | 13.1 (12.8, 13.4) | 2.2 (1.7, 2.7) | <0.001 | 0.38 |
| *Notes.* The inverted Nutri-Score scale (for foods included in this study) ranged from -12 to 30, with a lower score indicating a lower nutritional value and a higher score indicating a higher nutritional value. CI = confidence interval. Difference is experimental minus control. P-values calculated using linear regressions. To calculate the “Overall” Nutri-Score, we calculated each participant’s mean Nutri-Score across the three food categories, then used this value in the linear regression model. | | | | | |

| **Table S6.** Mean carbon footprint and differences by trial arm (n=2,149). | | | | | |
| --- | --- | --- | --- | --- | --- |
|  | **Experimental Arm**  **(n=1,074)** | **Control Arm**  **(n=1,075)** | **Difference (95% CI)** | **p-value** | **Cohen’s d** |
| **kg CO_2_-eq/100g** | **Adjusted Mean (95% CI)** | **Adjusted Mean (95% CI)** |  |  |  |
| Snack | 0.47 (0.37, 0.57) | 1.39 (1.29, 1.49) | -0.92 (-1.06, -0.78) | <0.001 | 0.56 |
| Protein | 0.79 (0.70, 0.88) | 1.95 (1.86, 2.05) | -1.16 (-1.29, -1.03) | <0.001 | 0.76 |
| Frozen | 0.33 (0.31, 0.34) | 0.52 (0.51, 0.54) | -0.20 (-0.22, -0.17) | <0.001 | 0.67 |
| **Overall** | 0.53 (0.48, 0.58) | 1.29 (1.24, 1.34) | -0.76 (-0.83, -0.69) | <0.001 | 0.93 |
| *Notes.* kg CO_2_-eq/100g = kilograms of carbon dioxide equivalents per 100 grams of food. Values are from the dataFRIENDS database and range from 0.00 to 5.42 kg CO_2_-eq/100g (13, 14). CI = confidence interval. Difference is experimental minus control. P-values calculated using linear regressions. To calculate the “Overall” carbon footprint, we calculated each participant’s mean carbon footprint across the three food categories, then used this value in the linear regression model. | | | | | |

| **Table S7.** Mean carbon footprint and differences prior to the peer comparison messages and swap recommendations by trial arm (n=2,149). | | | | | |
| --- | --- | --- | --- | --- | --- |
|  | **Experimental Arm**  **(n=1,074)** | **Control Arm**  **(n=1,075)** | **Difference (95% CI)** | **p-value** | **Cohen’s d** |
| **kg CO_2_-eq/100g** | **Adjusted Mean (95% CI)** | **Adjusted Mean (95% CI)** |  |  |  |
| Snack | 0.74 (0.63, 0.84) | 1.39 (1.29, 1.49) | -0.65 (-0.80, -0.50) | <0.001 | 0.37 |
| Protein | 1.26 (1.15, 1.36) | 1.95 (1.86, 2.05) | -0.70 (-0.84, -0.55) | <0.001 | 0.40 |
| Frozen | 0.39 (0.37, 0.41) | 0.52 (0.51, 0.54) | -0.13 (-0.16, -0.11) | <0.001 | 0.43 |
| **Overall** | 0.79 (0.74, 0.85) | 1.29 (1.24, 1.34) | -0.49 (-0.57, -0.42) | <0.001 | 0.38 |
| *Notes.* kg CO_2_-eq/100g = kilograms of carbon dioxide equivalents per 100 grams of food. Values are from the dataFRIENDS database and range from 0.00 to 5.42 kg CO_2_-eq/100g (13, 14). CI = confidence interval. Difference is experimental minus control. P-values calculated using linear regressions. To calculate the “Overall” carbon footprint, we calculated each participant’s mean carbon footprint across the three food categories, then used this value in the linear regression model. | | | | | |

| **Table S8.** Acceptability of intervention components. | | | | |  |
| --- | --- | --- | --- | --- | --- |
|  | **Experimental Arm**  **(n=1,074)** | **Control Arm**  **(n=1,075)** | **Difference (95% CI)** | **p-value** | **Cohen’s d** |
| **Intervention components** | **Adjusted Mean (95% CI)** | **Adjusted Mean (95% CI)** |  |  |  |
| Climate-impact labels | 3.7 (3.6, 3.8) | 3.6 (3.5, 3.6) | 0.1 (0.0, 0.2) | 0.01 | 0.11 |
| Social comparison messages | 3.5 (3.4, 3.5) | 3.3 (3.2, 3.3) | 0.2 (0.1, 0.3) | <0.001 | 0.17 |
| Swap recommendations | 3.5 (3.4, 3.5) | 3.4 (3.3, 3.5) | 0.1 (0.0, 0.2) | 0.31 | 0.04 |

**References**

1. Scientific Community of the Nutri-Score. *Update of the Nutri-Score algorithm* [Internet]. 2022; Available from: <https://www.aesan.gob.es/AECOSAN/docs/documentos/Nutri_Score/2022_main_algorithm_report_update_FINAL.pdf>.

2. Santé publique France. *Nutri-Score* [Internet]. 2021; Available from: <https://www.santepubliquefrance.fr/en/nutri-score>.

3. Farvid MS, Sidahmed E, Spence ND, Mante Angua K, Rosner BA, Barnett JB. Consumption of red meat and processed meat and cancer incidence: a systematic review and meta-analysis of prospective studies. Eur J Epidemiol. 2021;36(9):937-51. doi: 10.1007/s10654-021-00741-9.

4. Grosso G, La Vignera S, Condorelli RA, Godos J, Marventano S, Tieri M, et al. Total, red and processed meat consumption and human health: an umbrella review of observational studies. Int J Food Sci Nutr. 2022;73(6):726-37. doi: 10.1080/09637486.2022.2050996.

5. Matthews K, Phelan J, Jones NA, Konya S, Marks R, Pratt BM, Coombs J, Bentley M. 2015 National Content Test Race and Ethnicity Analysis Report: A New Design for the 21st Century. U.S. Department of Commerce, Economics and Statistics Administration, U.S. Census Bureau, 2017.

6. Zoellner J, Estabrooks PA, Davy BM, Chen YC, You W. Exploring the theory of planned behavior to explain sugar-sweetened beverage consumption. J Nutr Educ Behav. 2012;44(2):172-7. doi: 10.1016/j.jneb.2011.06.010.

7. Green-Score. *Présentation* [Internet]. 2025; Available from: <https://docs.score-environnemental.com/>.

8. Vargas-Meza J, Jáuregui A, Contreras-Manzano A, Nieto C, Barquera S. Acceptability and understanding of front-of-pack nutritional labels: an experimental study in Mexican consumers. BMC Public Health. 2019;19(1):1751. doi: 10.1186/s12889-019-8108-z.

9. Williams VF, Smith AA, Villanti AC, Rath JM, Hair EC, Cantrell J, Teplitskaya L, Vallone DM. Validity of a Subjective Financial Situation Measure to Assess Socioeconomic Status in US Young Adults. J Public Health Manag Pract. 2017;23(5):487-95. doi: 10.1097/phh.0000000000000468.

10. Princeton Survey Research Associates International for the Pew Research Center’s Internet & American Life Project. *Civic Engagement Tracking Survey 2012* [Internet]. Pew Research Center; 2012; Available from: <https://www.pewresearch.org/internet/wp-content/uploads/sites/9/2013/04/SurveyQuestions_CivicEngagement.pdf>.

11. *Diet Behavior and Nutrition - DBQ* [Internet]. Centers for Disease Control and Prevention, National Center for Health Statistics; 2020; Available from: <https://wwwn.cdc.gov/nchs/data/nhanes/2019-2020/questionnaires/DBQ_K.pdf>.

12. Haws KL, Winterich KP, Naylor RW. Seeing the world through GREEN-tinted glasses: Green consumption values and responses to environmentally friendly products. Journal of Consumer Psychology. 2014;24(3):336-54. doi: 10.1016/j.jcps.2013.11.002.

13. Heller MC, Willits-Smith A, Meyer R, Keoleian GA, Rose D. Greenhouse gas emissions and energy use associated with production of individual self-selected US diets. Environ Res Lett. 2018;13(4):044004. doi: 10.1088/1748-9326/aab0ac.

14. Rose D, Heller MC, Willits-Smith AM, Meyer RJ. Carbon footprint of self-selected US diets: nutritional, demographic, and behavioral correlates. Am J Clin Nutr. 2019;109(3):526-34. doi: 10.1093/ajcn/nqy327.
